# Association of *NOD1*, *CXCL16*, *STAT6* and *TLR4* gene polymorphisms with Malaysian patients with Crohn’s disease

**DOI:** 10.7717/peerj.1843

**Published:** 2016-03-31

**Authors:** Kek Heng Chua, Jin Guan Ng, Ching Ching Ng, Ida Hilmi, Khean Lee Goh, Boon Pin Kee

**Affiliations:** 1 Department of Biomedical Science, Faculty of Medicine, Universiti Malaya, Kuala Lumpur, Malaysia; 2 Division of Genetics and Molecular Biology, Institute of Biological Science, Faculty of Science, Universiti Malaya, Kuala Lumpur, Malaysia; 3 Division of Gastroenterology and Hepatology, Department of Medicine, Faculty of Medicine, Universiti Malaya, Kuala Lumpur, Malaysia

**Keywords:** *NOD1*, *CXCL16*, *STAT6*, *TLR4*, SNP, Crohn’s disease

## Abstract

Crohn’s disease (CD) is a prominent type of inflammatory bowel disease (IBD) that can affect any part of the gastrointestinal tract. CD is known to have higher prevalence in the Western countries, but the number of cases has been increasing in the past decades in Asia, including Malaysia. Therefore, there is a need to investigate the underlining causes of CD that may shed light on its prevention and treatment. In this study, genetic polymorphisms in *NOD1* (rs2075820), *CXCL16* (rs2277680), *STAT6* (rs324015) and *TLR4* (rs4986791) genes were examined in a total of 335 individuals (85 CD patients and 250 healthy controls) with PCR-RFLP approach. There was no significant association observed between *NOD1* rs2075820 and *STAT6* rs324015 with the onset of CD in the studied cohort. However, the G allele of *CXCL16* rs2277680 was found to have a weak association with CD patients (*P* = 0.0482; *OR* = 1.4310). The *TLR4* rs4986791 was also significantly associated to CD. Both the homozygous C genotype (*P* = 0.0029; *OR* = 0.3611) and C allele (*P* = 0.0069; *OR* = 0.4369) were observed to confer protection against CD. On the other hand, the heterozygous C/T genotype was a risk genotype (*P* = 0.0015; *OR* = 3.1392). Further ethnic-stratified analysis showed that the significant associations in *CXCL16* rs2277680 and *TLR4* rs4986791 were accounted by the Malay cohort. In conclusion, the present study reported two CD-predisposing loci in the Malay CD patients. However, these loci were not associated to the onset of CD in Chinese and Indian patients.

## Introduction

Both Crohn’s disease (CD) and ulcerative colitis (UC), the major sub-types of inflammatory bowel disease (IBD), are regarded as important global health issue, especially in the Western countries (Hilmi, Tan & Goh, 2006). Despite the similarities of some of the clinical symptoms shared by CD and UC, they can be differentiated by the affected locations. CD patients often suffer from multiple inflammation along the entire gastrointestinal tract, including mouth and anus. Occasionally, CD also inflicts complications that involve other organs, such as eyes, joints, blood, skin, and endocrine system. On the other hand, UC displays relatively restricted clinical manifestations, which are always localised at colon and rectum. To date, there is yet a cure for both CD and UC. Treatments and medical procedures are given to patients mainly for symptomatic relief and maintaining remission, which may also come with the cost of side effects, such as nausea and skin rashes (Sandborn et al., 2007).

Despite numerous extensive research studies that were conducted to find the etiology of CD in the past decades, the exact cause of CD remains inconclusive. However, it is generally accepted that, like other autoimmune diseases, the onset of CD could be triggered by the interaction of two major factors, i.e., genetic and environment (Baumgart & Carding, 2007). The increased disease concordance rate in monozygotic twins and higher risk of disease development in siblings of affected individuals highlight the importance of genetic predisposition in the disease development (Orholm et al., 2000). A number of genes have been identified through large scale Genome Wide Association Studies (GWAS) and meta-analysis to have significant linkage with the development of CD in the past (Duerr et al., 2006; Hampe et al., 2007). However, none of these genes was found as the sole contributor to the disease, suggesting polygenic traits in the disease development and progression.

CD are commonly observed in the Western countries, e.g. northern Europe, United Kingdom, and North America (Hilmi, Tan & Goh, 2006). The prevalence rate of CD has been relatively low in developing countries in Asia. Until recently, several studies had reported a rising trend of CD in Sri Lanka, Hong Kong, Japan, and Singapore (Morita et al., 1995; Lee et al., 2000; Niriella et al., 2010). Scientists postulate that this phenomenon might result from the adoption of modern lifestyles from Western counterparts (Thia et al., 2008). The overall prevalence rates of CD in Malaysia are 26 per 100,000 person (Tan & Goh, 2005; Hilmi, Tan & Goh, 2006). The prevalence rate differs among the three main ethnic groups that make up the multiracial society of Malaysia, i.e., Malay, Chinese, and Indian. CD appears most commonly in Indians, followed by Chinese and Malays, with prevalence rates of 52.6, 26.9, and 9.2 per 100,000 person respectively (Tan & Goh, 2005; Hilmi, Tan & Goh, 2006). Some gene and CD association studies have also been conducted previously (Chua et al., 2011; Chua et al., 2012; Chua et al., 2015); however, the actual genetics architecture of CD in the Malaysian populations still remains unclear.

Nucleotide-binding Oligomerization Domain-containing 1 (*NOD1*) protein is a member of NOD-like receptor (NLR) family. It is involved in the activation of NF-*κ*B and caspase-9 that are important to cellular apoptosis and immune response, respectively (Inohara et al., 1999). *NOD1* recognises the invasion of bacteria by reacting with a unique dipeptide, *γ*-D-glutamyl-meso-diaminopimelic acid (iE-DAP), that is present in the bacterial peptidoglycan (Chamaillard et al., 2003). The gene that encodes *NOD1* protein is located on chromosome 7p14. Genetic variations in this gene have been associated to asthma and IBD (Hysi et al., 2005; McGovern et al., 2005). The *NOD*1 rs2075820 involves the replacement of glutamic acid (E) by lysine (K) at amino acid position-266. This SNP has been shown to increase the susceptibility of gastric mucosal inflammation following *Helicobacter pylori* infection, possibly due to altered plasma level of NF-*κ*B (Kara et al., 2010).

Chemokine (C-X-C motif) Ligand 16 (*CXCL16*) protein is produced by dendritic cells and splenic red pulp cells (Wilbanks et al., 2001). It is also expressed in macrophages. It interacts with CXC chemokine receptors 6 (CXCR6) during inflammation and recruits the CXCR6 expressing cells, such as naive CD8 cells, intraepithelial lymphocytes, natural killer T cells, activated CD8 and CD4 T cells, to the affected site (Matloubian et al., 2000). *CXCL16* is constantly expressed in human cells in two forms, i.e., membrane-bound and soluble forms. The membrane-bound *CXCL16* aids the adhesion of bacteria for phagocytosis, whereas the soluble form induces the migration of activated T-cells (Abel et al., 2004; Gough et al., 2004; Nakase et al., 2012). The gene for human *CXCL16* is on chromosome 17p13. Study has found that the serum level of soluble form of *CXCL16* was increased more than 10 times in CD patients compared to controls (Lehrke et al., 2008). Previous study has demonstrated the association of *CXCL16* Ala181Val (rs2277680) variant with severe disease phenotype in young CD patients (Seiderer et al., 2008).

Signal Transducer and Activator of Transcription 6 (*STAT6*) is a transcription factor in STAT family (Hamlin et al., 1999). Unlike other members in the family, STAT6 is activated by interleukin-4 (IL-4) through tyrosine phosphorylation. The phosphorylated STAT6 dimerizes and translocates to cell nucleus. It binds to specific DNA and activates the transcription of certain IL-4-induced genes, which leads to the initiation of humoral immunity respond by T-helper 2 (Th2) cell. Th2 cells promote the production of IgE and the proliferation of mast cells/eosinophils, leading to inflammation (Romagnani, 1999). The *STAT6* gene is located on chromosome 12q13. Studies had shown that the G2964A variation (rs324015) within its 3′ untranslated region (UTR) was related to familial CD (Klein et al., 2005).

Toll-Like Receptor 4 (*TLR4*) gene is located on chromosome 9q33. *TLR4* protein is highly conserved and functions as a mediator for production of cytokines in innate immune system. It also plays a vital role in pathogen recognition. *TLR4* senses the lipopolysaccharides (LPS) from Gram-negative bacteria. Binding of the bacterial LPS to *TLR4*, together with other accessory proteins such as LPS-binding protein, MD2, and CD14, activates the production of NF-*κ* B and other cytokines (Philpott & Girardin, 2004; Lavelle et al., 2010). Alteration of the protein structure, due to mutations or SNP, results in differential responsiveness to LPS and may induce massive inflammatory reaction. *TLR4* T399I (rs4986791) was related to weakened LPS response and other diseases, i.e., diabetes, rheumatoid arthritis, Alzheimer’s disease, renal and cardiovascular disease (Arbour et al., 2000; O’Neill, Bryant & Doyle, 2009). It was also found to associate with the development of CD and UC (Torok et al., 2004; Zouiten-Mekki et al., 2009).

In the present study, we aim to investigate the distribution of four SNPs in *NOD1* (rs2075820), *CXCL16* (rs2277680), *STAT6* (rs324015) and *TLR4* (rs4986791) genes in Malaysian patients with CD. We reported the association of alleles and genotypes of these SNPs with the onset of CD in the Malaysian population.

## Materials and Methods

### Subject recruitment

The studied samples consisted of 335 individuals from the three major ethnic groups in Malaysia, i.e., Malay, Chinese, and Indian, living around Kuala Lumpur city. A total of 85 CD patients (20 Malays, 27 Chinese, 38 Indians) and 250 (74 Malays, 86 Chinese, 90 Indians) healthy controls were recruited in this study. All patients were diagnosed at University Malaya Medical Centre (UMMC), Kuala Lumpur, Malaysia, based on Vienna classification (Table 1). The research methods in this study were approved by UMMC Ethics Review Board (MEC reference number: 472.55) and informed consents were obtained from all participants prior to blood sample collection.

**Table 1:** Clinical characteristics of CD patients in the present study according to the Vienna classification system.

Characteristics	Crohn’s disease (CD) (*N* = 85)	Control (*N* = 250)
Age^*^ (years)	42.0 ± 18.1	37.1 ± 11.2
Age at diagnosis^*^ (years)	29.2 ± 14.8	
Positive family history	None	
Location		
Terminal ileum (L1)	20 (23.5%)	
Colon (L2)	46 (54.1%)	
Ileocolon (L3)	11 (12.9%)	
Upper gastrointestinal (L4)	5 (5.9%)	
Phenotypes		
Non-stricturing non-penetrating (B1)	50 (58.8%)	
Stricturing (B2)	19 (22.4%)	
Penetrating (B3)	9 (10.6%)	

**Notes.**

* Mean ± standard deviation

### SNP genotyping

Blood sample was collected from each participant in an EDTA tube. Genomic DNA was extracted from all samples via a conventional phenol-chloroform extraction method (Chua et al., 2009). Post-extraction quality check was carried out with spectrophotometry to ensure high-quality DNA for the subsequent genotyping experiments.

SNP genotyping was carried out via polymerase chain reaction-restricted fragment length polymorphism (PCR-RFLP) method. The amplification of DNA regions covering the SNPs of interest was conducted with a standard PCR in a thermal cycler (Veriti; Life Technologies, Carlsbad, CA, USA). The final reaction mixture contained 1X *Taq* buffer, 0.375 µM of respective forward and reverse primers, 0.075 mM dNTP mix, 0.75 unit of *Taq* DNA polymerase, 100 ng of template DNA, and topped up to a final volume of 20 µl with sterile distilled water. A universal PCR cycling parameter was used: initial denaturation at 95 °C for 12 min, followed by 35 cycles of 94 °C for 30 s, 60 °C (*TLR4* rs4986791), 64 °C (*NOD1* rs2075820 and *CXCL16* rs2277680), or 68 °C (*STAT6* rs324015) for 30 s, and 72 °C for 30 s, final extension at 72 °C for 7 min. The amplified products were subjected to digestion with 5 unit of respective restriction enzymes at 37 °C for overnight. Primer sequences, amplicon sizes, and restriction enzymes used for SNP genotyping are summarised in Table 2. The digested products were applied on a 2.5% (w/v) agarose gel stained with ethidium bromide and visualised using a UV transilluminator (Figs. S1–S4). Allelic and genotypic distribution of the SNPs were done by direct counting.

**Table 2:** Primer sequences, amplicon sizes, and restriction enzymes used for the PCR-RFLP study.

SNP	Forward/Reverse primer (5′–3′)	Amplicon size (bp)	Restriction enzyme	Reference
*NOD1* rs2075820	TGAGACCATCTTCATCCTGG/	379	*Ava*I	Molnar et al., 2007
	CTTCCCACTGAGCAGGTTG			
*CXCL16* rs2277680	ACTCGTCCCAATGAAACCAC/	499	*Alu*I	Lundberg et al., 2005
	CCACAGCTTCATCTCCCACT			
*STAT6* rs324015	GAAGTTCAGGCTCTGAGAGAC/	159	*Hin1*I	Klein et al., 2005
	AGAATGGGCGGAGAAGCCT			
*TLR4* rs4986791	GGTTGCTGTTCTCAAAGTGATTTTGGGAGAA/	124	*Hinf*I	Torok et al., 2004
	GGAAATCCAGATGTTCTAGTTGTTCTAAGCC			

Validation of the genetic data generated from the PCR-RFLP method was conducted via a PCR-resequencing approach. Representative samples of each observed genotype for the four studied SNPs were selected and amplified in standard PCR reactions using SNP-specific primers. The amplified products were purified and subjected to DNA sequencing reaction. The genotype of each selected sample was determined based on the signal patterns at the particular SNP location in the electropherograms.

### Statistical analysis

The allelic and genotypic frequencies of these polymorphisms were calculated. Deviation from Hardy–Weinberg equilibrium was assessed via Arlequin V3.11 software (Excoffier & Lischer, 2010). The power of statistics was calculated with Quanto V1.2 (USC Biostats, Los Angeles, CA, USA), based on overall disease risk, allelic and genotypic frequencies. Fisher’s exact test, odds ratio (OR), and 95% confidence interval (CI) were analysed to correlate the polymorphisms to the onset of CD in Malaysian population. The significant level was set at *P* < 0.05. Correction of *P* values for multiple comparison was also calculated based on Bonferroni and False Discovery Rate (FDR) (Dunn, 1961; Benjamini & Hochberg, 1995).

## Results

### *NOD1* rs2075820 G/A polymorphism

No significant association was observed for rs2075820 G/A polymorphism in *NOD1* gene in the present study based on the allelic and genotypic data (Table 3). Both G and A alleles were evenly distributed in CD patients and controls. Although the homozygous A genotype was found two times higher in the controls (12.0%) compared to patients (5.9%), the distribution was not significant (*P* = 0.1498). Further statistical analysis was performed based on stratification of samples to the three main ethnic groups, i.e., Chinese, Malay, and Indian (Table 4). Despite the observation of over three times higher frequency of the homozygous A genotype in the Indian CD patients, no significant association was observed.

**Table 3:** Genotypic and allelic distribution of *NOD1* rs2075820 polymorphism in Malaysian Crohn’s disease (CD) patients and control individuals.

*NOD1* rs2075820	Frequency, *n* (%)			
	CD (*N* = 85)	Control (*N* = 250)	*P* value	OR (95% CI)
Genotype				
G/G	33 (38.8)	98 (39.2)	1.0000	0.9843 (0.5942–1.6305)
G/A	47 (55.3)	122 (48.8)	0.3174	1.2977 (0.7916–2.1274)
A/A	5 (5.9)	30 (12.0)	0.1498	0.4583 (0.1719–1.2220)
Allele				
G	113 (66.5)	318 (63.6)	0.5179	1.1346 (0.7862–1.6374)
A	57 (33.5)	182 (36.4)		0.8814 (0.6107–1.2720)

**Notes.**

OR odds ratio CI confidence interval

**Table 4:** Genotypic and allelic distribution of *NOD1* rs2075820 polymorphism in Chinese, Malays, and Indians.

Ethnic group		Frequency, *n* (%)		*P* value	OR (95% CI)
Chinese	Genotype	CD (*N* = 27)	Control (*N* = 86)		
	G/G	12 (44.4)	38 (44.2)	1.0000	1.0105 (0.4232–2.4127)
	G/A	14 (51.9)	41 (47.7)	0.8260	1.1820 (0.4975–2.8084)
	A/A	1 (3.7)	7 (8.1)	0.6775	0.4341 (0.0510–3.6955)
	Allele				
	G	38 (70.4)	117 (68.0)	0.8668	1.1165 (0.5735–2.1736)
	A	16 (29.6)	55 (32.0)		0.8957 (0.4601–1.7438)
Malay	Genotype	CD (*N* = 20)	Control (*N* = 74)		
	G/G	10 (50.0)	36 (48.6)	1.0000	1.0556 (0.3930–2.8350)
	G/A	8 (40.0)	30 (40.5)	1.0000	0.9778 (0.3569–2.6787)
	A/A	2 (10.0)	8 (10.8)	1.0000	0.9167 (0.1787–4.7011)
	Allele				
	G	28 (70.0)	102 (68.9)	0.5179	1.0523 (0.4918–2.2514)
	A	12 (30.0)	46 (31.1)		0.9503 (0.4442–2.0332)
Indian	Genotype	CD (*N* = 38)	Control (*N* = 90)		
	G/G	11 (28.9)	24 (26.6)	0.8298	1.1204 (0.4825–2.6017)
	G/A	25 (65.8)	51 (56.7)	0.4313	1.4706 (0.6679–3.2380)
	A/A	2 (5.3)	15 (16.7)	0.0949	0.2778 (0.0603–1.2803)
	Allele				
	G	47 (61.8)	99 (55.0)	0.3359	1.3260 (0.7665–2.2940)
	A	29 (38.2)	81 (45.0)		0.7281 (0.4191–1.2651)

**Notes.**

OR odds ratio CI confidence interval

**Table 5:** Genotypic and allelic distribution of *CXCL16* rs2277680 polymorphism in Malaysian Crohn’s disease (CD) patients and control individuals.

*CXCL16* rs2277680	Frequency, *n* (%)			
	CD (*N* = 85)	Control (*N* = 250)	*P* value	OR (95% CI)
Genotype				
A/A	23 (27.1)	89 (35.6)	0.1832	0.6711 (0.3895–1.1563)
A/G	41 (48.2)	122 (48.8)	1.0000	0.9776 (0.5974–1.5997)
G/G	21 (24.7)	39 (15.6)	0.0713	1.7752 (0.9745–3.2337)
Allele				
A	87 (51.2)	300 (60.0)	0.0482^*^	0.6988 (0.4925–0.9916)
G	83 (48.8)	200 (40.0)		1.4310 (1.0085–2.0306)

**Notes.**

* *P* < 0.05.

OR odds ratio CI confidence interval

### *CXCL16* rs2277680 A/G polymorphism

As shown in Table 5, the G allele of *CXCL16* rs2277680 was observed to have a weak association to the CD patients (*P* = 0.0482; *OR* = 1.4310). However, the significance was dismissed after the correction by Bonferroni and FDR. There was strong significant correlation observed for the genotypes, except the homozygous G genotype, which has a borderline *P* value of 0.0713. These observations suggest that the G allele may serve as a predisposing factor to CD in the Malaysian cohort. Our postulation was supported by ethnic-stratified analysis (Table 6). Significant associations were found in the Malay cohort for both G allele (*P* = 0.0306; *OR* = 2.2227) and homozygous G genotype (*P* = 0.0154; *OR* = 4.4423). However, the statistical power computed by Quanto software was only 0.5931 for the association. In addition, the corrected *P* values for both G allele and homozygous G genotype were not significant (*P* > 0.05). On the other hand, no significant association was observed in Chinese and Indian CD patients.

**Table 6:** Genotypic and allelic distribution of *CXCL16* rs2277680 polymorphism in Chinese, Malays, and Indians.

Ethnic group		Frequency, *n* (%)		*P* value	OR (95% CI)
Chinese	Genotype	CD (*N* = 27)	Control (*N* = 86)		
	A/A	10 (37.0)	36 (41.8)	0.8227	0.8170 (0.3352–1.9913)
	A/G	11 (40.8)	41 (47.7)	0.6588	0.7546 (0.3141–1.8130)
	G/G	6 (22.2)	9 (10.5)	0.1890	2.4444 (0.7817–7.6443)
	Allele				
	A	31 (57.4)	113 (65.7)	0.3304	0.7037 (0.3769–1.3141)
	G	23 (42.6)	59 (34.3)		1.4210 (0.7609–2.6536)
Malay	Genotype	CD (*N* = 20)	Control (*N* = 74)		
	A/A	4 (20.0)	26 (35.1)	0.2812	0.4615 (0.1397–1.5249)
	A/G	9 (45.0)	40 (54.1)	0.6149	0.6955 (0.2578–1.8764)
	G/G	7 (35.0)	8 (10.8)	0.0154^*^	4.4423 (1.3707–14.3976)
	Allele				
	A	17 (42.5)	92 (62.2)	0.0306^*^	0.4499 (0.2213–0.9146)
	G	23 (57.5)	56 (37.8)		2.2227 (1.0933–4.5186)
Indian	Genotype	CD (*N* = 38)	Control (*N* = 90)		
	A/A	9 (23.7)	27 (30.0)	0.5247	0.7241 (0.3024–1.7341)
	A/G	21 (55.3)	41 (45.6)	0.3390	1.4763 (0.6889–3.1639)
	G/G	8 (21.1)	22 (24.4)	0.8204	0.8242 (0.3297–2.0604)
	Allele				
	A	39 (51.3)	95 (52.8)	0.8913	0.9431 (0.5515–1.6129)
	G	37 (48.7)	85 (47.2)		1.0603 (0.6200–1.8134)

**Notes.**

* *P* < 0.05.

OR odds ratio CI confidence interval

**Table 7:** Genotypic and allelic distribution of *STAT6* rs324015 polymorphism in Malaysian Crohn’s disease (CD) patients and control individuals.

*STAT6* rs324015	Frequency, *n* (%)			
	CD (*N* = 85)	Control (*N* = 250)	*P* value	OR (95% CI)
Genotype				
A/A	14 (16.5)	53 (21.2)	0.4328	0.7329 (0.3832–1.4017)
A/G	42 (49.4)	115 (46.0)	0.6161	1.1466 (0.7006–1.8765)
G/G	29 (34.1)	82 (32.8)	0.8940	1.0610 (0.6306–1.7853)
Allele				
A	70 (41.2)	221 (44.2)	0.5310	0.8837 (0.6210–1.2575)
G	100 (58.8)	279 (55.8)		1.1316 (0.7952–1.6103)

**Notes.**

OR odds ratio CI confidence interval

**Table 8:** Genotypic and allelic distribution of *STAT6* rs324015 polymorphism in Chinese, Malays, and Indians.

Ethnic group		Frequency, *n* (%)		*P* value	OR (95% CI)
Chinese	Genotype	CD (*N* = 27)	Control (*N* = 86)		
	A/A	7 (25.9)	24 (27.9)	1.0000	0.9042 (0.3389–2.4122)
	A/G	15 (55.6)	36 (41.9)	0.2689	1.7361 (0.7261–4.1508)
	G/G	5 (18.5)	26 (30.2)	0.3241	0.5245 (0.1791–1.5361)
	Allele				
	A	29 (53.7)	84 (48.8)	0.6401	1.2152 (0.6585–2.2428)
	G	25 (46.3)	88 (51.2)		0.8229 (0.4459–1.5187)
Malay	Genotype	CD (*N* = 20)	Control (*N* = 74)		
	A/A	2 (10.0)	16 (21.6)	0.3437	0.4028 (0.0844–1.9210)
	A/G	11 (55.0)	31 (41.9)	0.3213	1.6953 (0.6270–4.5839)
	G/G	7 (35.0)	27 (36.5)	1.0000	0.9373 (0.3334–2.6350)
	Allele				
	A	15 (37.5)	63 (42.6)	0.5126	0.8095 (0.6585–2.2428)
	G	25 (62.5)	85 (57.4)		1.2353 (0.6023–2.5335)
Indian	Genotype	CD (*N* = 38)	Control (*N* = 90)		
	A/A	5 (13.2)	13 (14.4)	1.0000	0.8974 (0.2960–2.7207)
	A/G	16 (42.1)	48 (53.3)	0.3335	0.6364 (0.2959–1.3684)
	G/G	17 (44.7)	29 (32.2)	0.2267	1.7028 (0.7826–3.7050)
	Allele				
	A	26 (34.2)	74 (41.1)	0.3286	0.7449 (0.4258–1.3030)
	G	50 (65.8)	106 (58.9)		1.3425 (0.7675–2.3485)

**Notes.**

OR odds ratio CI confidence interval

### *STAT6* rs324015 A/G polymorphism

Similar to *NOD1* rs2075820, there was no significant association observed in CD patients for both allelic and genotypic distribution (Tables 7 and 8). The A and G alleles were found almost equally in patients and control group. Although the homozygous A genotype present in higher percentage in the control individuals, and it was over two times higher in the Malay healthy controls than patients, no significant relationship was seen in Chinese, Malays, and Indian.

### *TLR4* rs4986791 C/T polymorphism

There were strong associations observed for the *TLR4* rs4986791 with the Malaysian CD patients (Table 9). The C allele (*P* = 0.0069; OR = 0.4369) and homozygous C genotype (*P* = 0.0029; OR = 0.3611) were found to confer protective effect against the onset of CD. On the other hand, the heterozygous C/T genotype present with higher percentage in CD patients (22.4%) than controls (8.4%) and significant association was observed (*P* = 0.0015; OR = 3.1392). These significant associations were supported by high statistical power as calculated by Quanto based on the sample size, i.e., 0.9691 and 0.9993 for homozygous C genotype and heterozygous C/T genotype respectively. Further stratification analysis showed that these significance were contributed by the Malay ethnic group, but not Chinese and Indian (Table 10). The C allele (*P* = 0.0003; OR = 0.0646) and homozygous C genotype (*P* = 0.0002; OR = 0.0516; Quanto = 0.8807) were found to be protective in the Malays. In contrast, the heterozygous C/T genotype was significantly associated to Malay CD patients (*P* = 0.0002; OR = 19.3846; Quanto = 0.8897). Interestingly, it appeared to be monomorphic in the Chinese cohort, where all individuals were homozygous C genotype. The homozygous T genotype was only seen in Indian, with less than 5%. All the significant associations observed in *TLR4* rs4986791 polymorphisms remain significant after correction by Bonferroni and FDR.

**Table 9:** Genotypic and allelic distribution of *TLR4* rs4986791 polymorphism in Malaysian Crohn’s disease (CD) patients and control individuals.

*TLR4* rs4986791	Frequency, *n* (%)			
	CD (*N* = 85)	Control (*N* = 250)	*P* value	OR (95% CI)
Genotype				
C/C	65 (76.4)	225 (90.0)	0.0029^*^	0.3611 (0.1886–0.6914)
C/T	19 (22.4)	21 (8.4)	0.0015^*^	3.1392 (1.5931–6.1859)
T/T	1 (1.2)	4 (1.6)	1.0000	0.7321 (0.0807–6.6423)
Allele				
C	149 (87.6)	471 (94.2)	0.0069^*^	0.4369 (0.2419–0.7890)
T	21 (12.4)	29 (5.8)		2.2891 (1.2676–4.1338)

**Notes.**

* *P* < 0.05.

OR odds ratio CI confidence interval

**Table 10:** Genotypic and allelic distribution of *TLR4* rs4986791 polymorphism in Chinese, Malays, and Indians.

Ethnic group		Frequency, *n* (%)		*P* value	OR (95% CI)
Chinese	Genotype	CD (*N* = 27)	Control (*N* = 86)		
	C/C	27 (100.0)	86 (100.0)	1.0000	N/A
	C/T	0 (0.0)	0 (0.0)	1.0000	N/A
	T/T	0 (0.0)	0 (0.0)	1.0000	N/A
	Allele				
	C	54 (100.0)	172 (100.0)	1.0000	N/A
	T	0 (0.0)	0 (0.0)		N/A
Malay	Genotype	CD (*N* = 20)	Control (*N* = 74)		
	C/C	13 (65.0)	72 (97.3)	0.0002^*^	0.0516 (0.0096–0.2765)
	C/T	7 (35.0)	2 (2.7)	0.0002^*^	19.3846 (3.6170–103.8874)
	T/T	0 (0.0)	0 (0.0)	1.0000	N/A
	Allele				
	C	33 (82.5)	146 (98.6)	0.0003^*^	0.0646 (0.0128–0.3251)
	T	7 (17.5)	2 (1.4)		15.4848 (3.0759–77.9549)
Indian	Genotype	CD (*N* = 38)	Control (*N* = 90)		
	C/C	25 (65.8)	67 (74.4)	0.3901	0.6602 (0.2906–1.4999)
	C/T	12 (31.6)	19 (21.1)	0.2592	1.7247 (0.7364–4.0392)
	T/T	1 (2.6)	4 (4.4)	1.0000	0.5811 (0.0628–5.3769)
	Allele				
	C	62 (81.6)	153 (85.0)	0.5760	0.7815 (0.7815–1.5892)
	T	14 (18.4)	27 (15.0)		1.2796 (0.6292–2.6020)

**Notes.**

* *P* < 0.05.

OR odds ratio CI confidence interval

### Validation study and Hardy–Weinberg equilibrium

The accuracy of the present PCR-RFLP study was validated by PCR-resequencing approach. Fifteen representative samples from each genotype (except for the only five homozygous T genotype for *TLR4* gene) was selected and amplified using specific primer sets in a conventional PCR. The amplicons were purified and subjected to bi-directional sequencing reactions. The genotype of all examined samples were determined based on the fluorescence signal on electropherograms (Figs. S5–S8). There was no discrepancy observed between the genotype determined by PCR-RFLP and PCR-resequencing methods. Hardy–Weinberg equilibrium was also computed for control groups of all three ethnic groups (Chinese, Malay, and Indian) and no deviation from the equilibrium was reported.

## Discussion

The investigation of *NOD1* rs2075820 in Malaysian CD patients showed that there was no significant association between the SNP with the onset of CD. Our result is in contrast to a genetic study conducted on Hungarian population, where the A allele was significantly observed in CD patients (Molnar et al., 2007). The rs2075820 presents in an evolutionary conserved region that consists of 171 amino acids. It interacts with a number of other domains such as CARD, WD40 repeat and LRR. It functions to hydrolyze ATP or GTP (Koonin & Aravind, 2000). The A allele was suggested to decrease the helix-formatting potential and affect the binding affinity of ATP or GTP to the domain (Molnar et al., 2007). *NOD1* polymorphisms were also often linked to inflammatory reaction following *Helicobacter pylori* infection, such as duodenal ulcer and gastritis (Hofner et al., 2007; Kara et al., 2010). Therefore, *NOD1* polymorphisms may not be related directly to the onset of CD, but through a complicated mechanism that is triggered by infection.

The *CXCL16* rs2277680 was significantly associated to CD patients in the Malaysian cohort. Overall, the G allele was found to have a weak predisposing effect on CD. When the data was analyzed according to ethnic group, significant association was only observed in the Malays. The G allele and homozygous G genotype carriers exerted two- and four-fold higher risk to develop CD in the Malay cohort. The statistical power of the sample size and corrected *P* values however, did not support the association. Therefore, more Malay CD patients could be recruited in future study to confirm this observation. There was no significant association established between the G allele with Chinese and Indian CD patients. This could be due to the difference in genetic background of these ethnic groups. Increased serum level of *CXCL16* has been reported in CD patients, suggesting a pro-inflammatory effect of *CXCL16* through the stimulation of C-reactive protein (Lehrke et al., 2008). The *CXCL16* rs2277680 involves the substitution of Alanine by Valine, which may enhance the efficiency of *CXCL16* protein and lead to the development of CD. The SNP was also reported to have no association to the susceptibility of CD in Caucasian population (Seiderer et al., 2008). However, it was shown to influence the clinical development of the disease, such as early age of onset.

The *STAT6* gene is situated well within *IBD2* susceptibility locus on chromosome 12. It is also one of the components in the pathway that triggers T-cell differentiation in inflammatory responses. Therefore, it may be a potential predisposing gene to the development of CD. In the present study, we did not observe significant association of rs324015 polymorphism, located in the 3′ UTR region of *STAT6*, with the establishment of CD in Malaysian cohort. Similar findings were also reported in Caucasian patients with CD from the Netherlands (De Jong et al., 2003; Xia et al., 2003). Interestingly, the G allele and homozygous G genotype were found significantly increased in CD patients without any variation in *CARD15* gene (Klein et al., 2005). The relationship of *STAT6* rs324015 with Asthma was also studied extensively but thus far, no significant association has been reported (Li et al., 2013; Qian et al., 2014). Based on these genetic studies, it is plausible to suggest that *STAT6* may not have a leading role in the development of inflammatory diseases.

In the overall Malaysian cohort, the *TLR4* rs4986791 polymorphism was shown to have very strong association to CD. The homozygous C genotype confers protection against the disease, whereas the heterozygous C/T carriers were seen to have three-fold higher chance to contract CD. Stratification analysis revealed that these associations were actually attributed to the Malay ethnic group, which showed more stringent levels of significance (*P* < 0.0003). Computation via Quanto software indicates that the sample size is sufficient to express high statistical power for the association analysis. In addition, the corrected *P* values for all associations remain significant. These further strengthen the association of the *TLR4* polymorphisms with the onset of CD in the Malay population. However, there was no significant association observed in the Chinese and Indian cohorts. It is interesting to note that the distribution of *TLR4* rs4986791 varied greatly in our study. It was monomorphic in the Chinese, where only homozygous C genotype was detected. Only two genotypes, homozygous C and heterozygous C/T, were seen in the Malays, and all three genotypes were observed in Indians. This may due to the discrepancy in genetic background of different ethnic groups and provide a likely explanation to many controversial case-control studies of *TLR4* rs4986791 on diverse populations. Many genetic studies could not establish the link between *TLR4* rs4986791 polymorphism and IBD patients from various Asian and Western countries (Browning et al., 2007). Several meta-analysis studies however, concluded that the T allele increases the risk of CD and/or UC, especially in Caucasian populations (Shen et al., 2010; Senhaji et al., 2014; Cheng et al., 2015). The T allele was suggested to impact the *TLR4* protein by affecting the transcription and expression of the gene (Cheng et al., 2015).

In conclusion, we reported significant associations of two loci (*CXCL16* rs2277680 and *TLR4* rs4986791) with Malay CD patients in Malaysia. However, there was no significant association observed for Chinese and Indian patients. A limitation of the current study was the small sample size of the CD patient cohort, particularly for the Malay ethnic group. Another limitation was the unknowing sub-ethnicity stratification within each of the three studied ethnic groups that may lead to ambiguous associations.

## Supplemental Information

10.7717/peerj.1843/supp-1 Figure S1 Separation of *Ava*I-digested PCR amplicon on 2.5% (w/v) native agarose gel for *NOD1* rs2075820 genotyping study L1: 100 bp DNA marker (GeneRuler, Thermo Scientific); L2: Undigested PCR product; L3: Homozygous A/A; L4: Heterozygous A/G; L5: Homozygous G/G; L6: Non-template control.

10.7717/peerj.1843/supp-2 Figure S2 Separation of *Alu*I-digested PCR amplicon on 2.5% (w/v) native agarose gel for *CXCL16* rs2277680 genotyping study L1: 50 bp DNA marker (Mini Sizer, Norgen Biotek Corp.); L2: Undigested PCR product; L3: Homozygous A/A; L4: Heterozygous A/G; L5: Homozygous G/G; L6: Non-template control.

10.7717/peerj.1843/supp-3 Figure S3 Separation of *Hin1*I-digested PCR amplicon on 2.5% (w/v) native agarose gel for *STAT6* rs324015 genotyping study L1: 50 bp DNA marker (GeneRuler, Thermo Scientific); L2: Undigested PCR product; L3: Homozygous A/A; L4: Heterozygous A/G; L5: Homozygous G/G; L6: Non-template control.

10.7717/peerj.1843/supp-4 Figure S4 Separation of *Hinf*I-digested PCR amplicon on 2.5% (w/v) native agarose gel for *TLR4* rs4986791 genotyping study L1: 50 bp DNA marker (GeneRuler, Thermo Scientific); L2: Undigested PCR product; L3: Homozygous C/C; L4: Heterozygous C/T; L5: Homozygous T/T; L6: Non-template control.

10.7717/peerj.1843/supp-5 Figure S5 Electropherogram of *NOD1* rs2075820 for validation study (A) Homozygous G/G (B) Homozygous A/A (C) Heterozygous G/A.

10.7717/peerj.1843/supp-6 Figure S6 Electropherogram of *CXCL16* rs2277680 for validation study (A) Homozygous A/A (B) Homozygous G/G (C) Heterozygous A/G.

10.7717/peerj.1843/supp-7 Figure S7 Electropherogram of *STAT6* rs324015 for validation study (A) Homozygous A/A (B) Homozygous G/G (C) Heterozygous A/G.

10.7717/peerj.1843/supp-8 Figure S8 Electropherogram of *TLR4* rs4986791 for validation study (A) Homozygous C/C (B) Homozygous T/T (C) Heterozygous C/T.

10.7717/peerj.1843/supp-9 Supplemental Information 9 Data S1

## Additional Information and Declarations

### Data Availability

The following information was supplied regarding data availability: Raw data has been uploaded as Supplemental Information.
