# Supplementary figures and images for: Association of NOD1, CXCL16, STAT6 and TLR4 gene polymorphisms with Malaysian patients with Crohn’s disease
Source: PeerJ. 2016 Mar 31;4:e1843. doi: 10.7717/peerj.1843 (PMC4824893; doi:10.7717/peerj.1843)

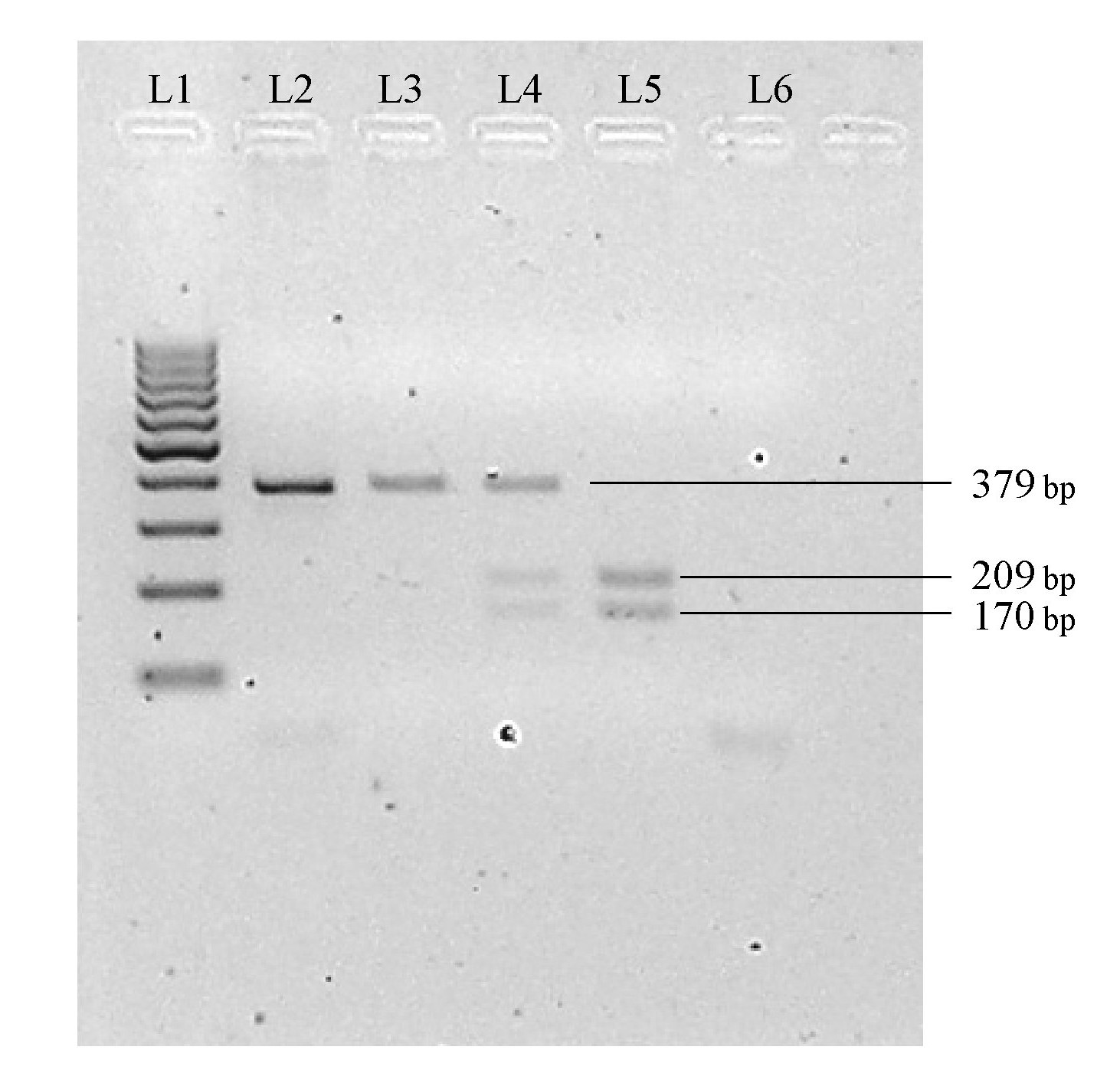

Supplement: Figure S1 — L1: 100 bp DNA marker (GeneRuler, Thermo Scientific); L2: Undigested PCR product; L3: Homozygous A/A; L4: Heterozygous A/G; L5: Homozygous G/G; L6: Non-template control. [file peerj-04-1843-s001.jpg]

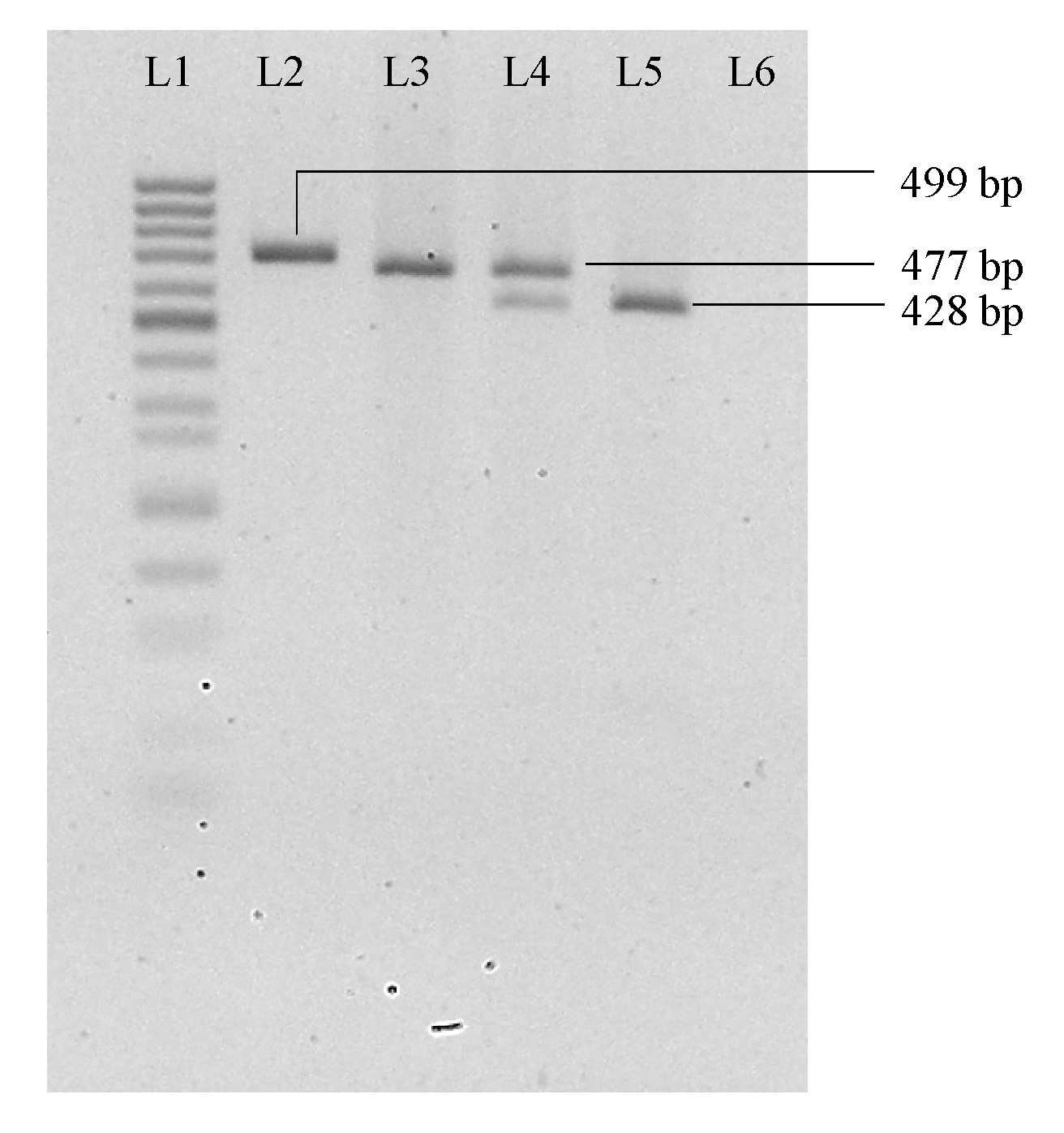

Supplement: Figure S2 — L1: 50 bp DNA marker (Mini Sizer, Norgen Biotek Corp.); L2: Undigested PCR product; L3: Homozygous A/A; L4: Heterozygous A/G; L5: Homozygous G/G; L6: Non-template control. [file peerj-04-1843-s002.jpg]

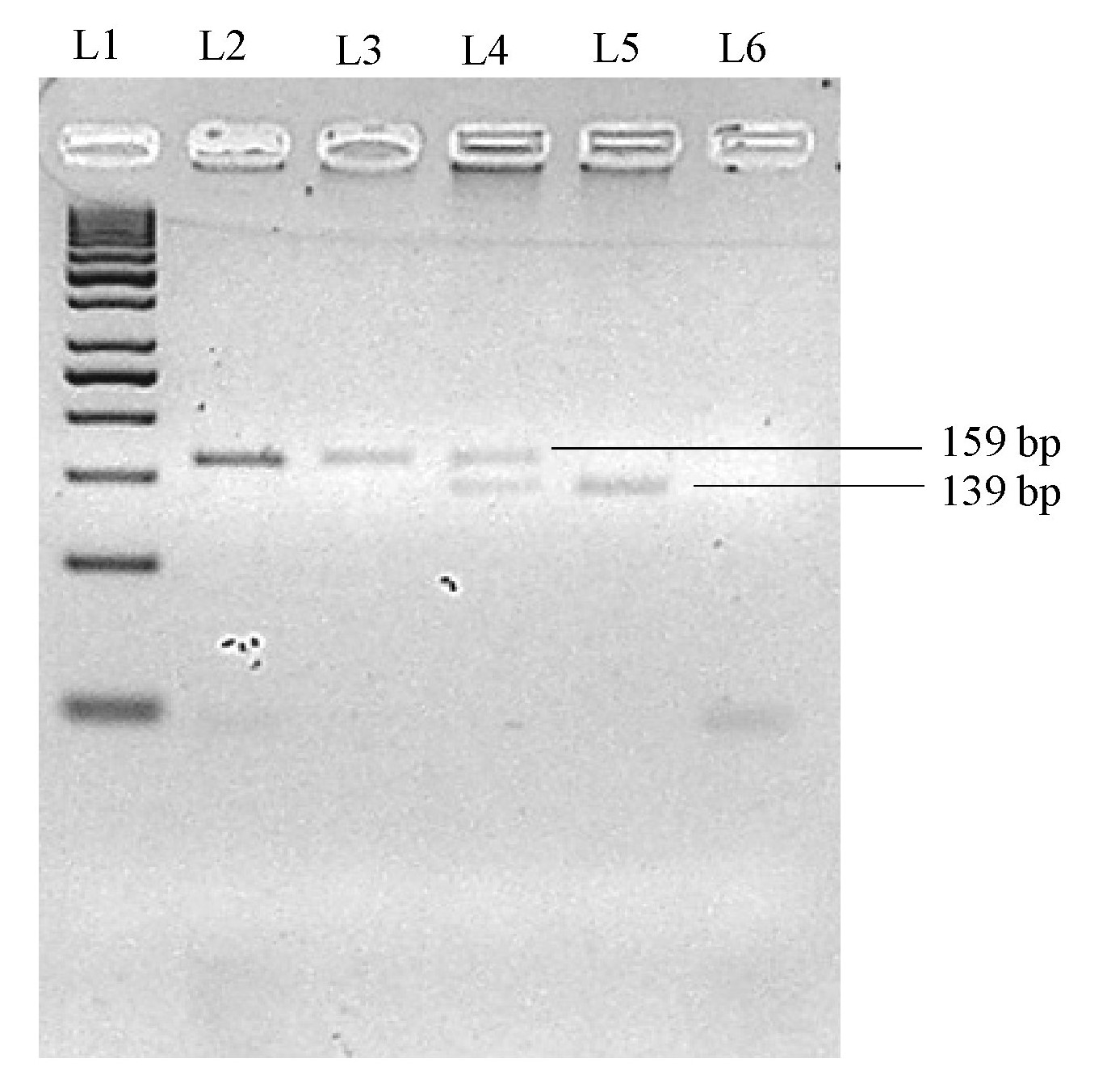

Supplement: Figure S3 — L1: 50 bp DNA marker (GeneRuler, Thermo Scientific); L2: Undigested PCR product; L3: Homozygous A/A; L4: Heterozygous A/G; L5: Homozygous G/G; L6: Non-template control. [file peerj-04-1843-s003.jpg]

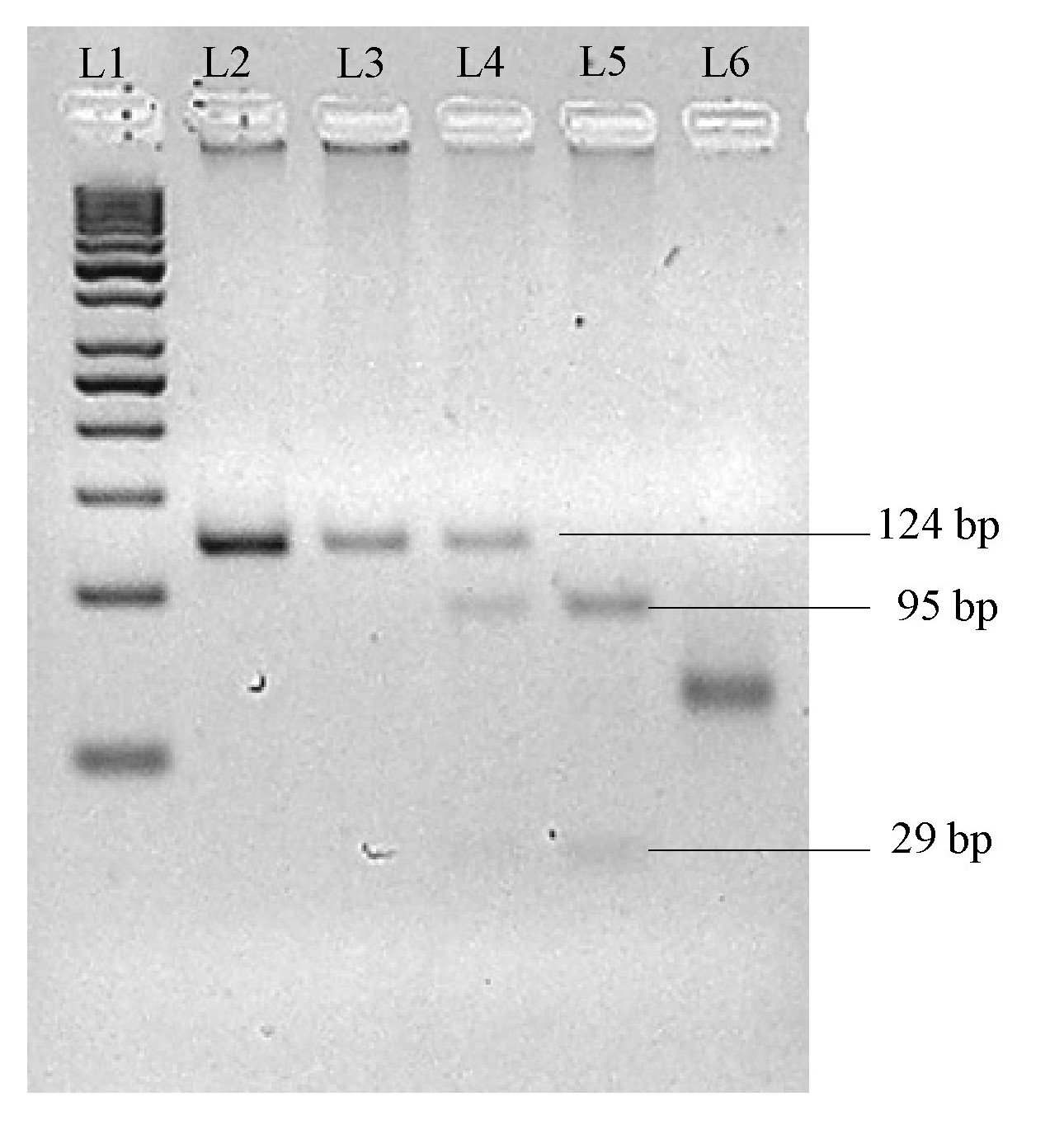

Supplement: Figure S4 — L1: 50 bp DNA marker (GeneRuler, Thermo Scientific); L2: Undigested PCR product; L3: Homozygous C/C; L4: Heterozygous C/T; L5: Homozygous T/T; L6: Non-template control. [file peerj-04-1843-s004.jpg]

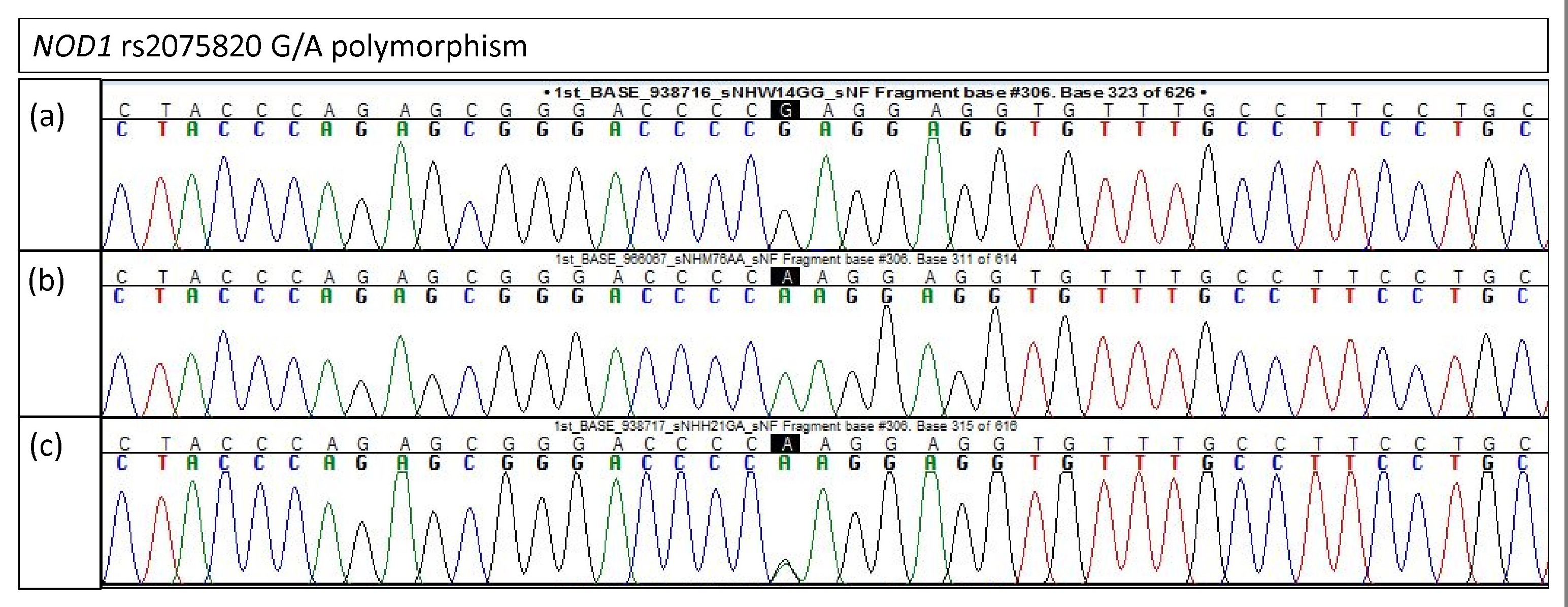

Supplement: Figure S5 — (A) Homozygous G/G (B) Homozygous A/A (C) Heterozygous G/A. [file peerj-04-1843-s005.jpg]

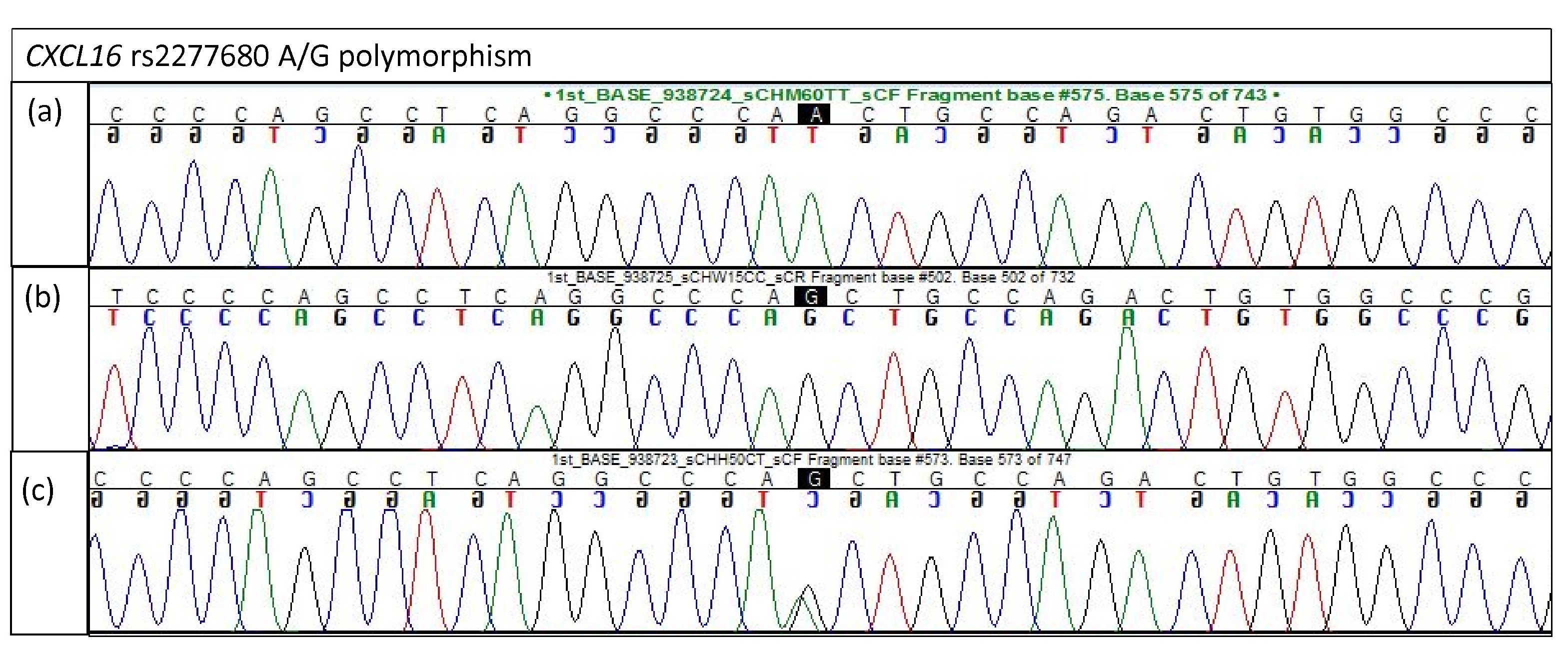

Supplement: Figure S6 — (A) Homozygous A/A (B) Homozygous G/G (C) Heterozygous A/G. [file peerj-04-1843-s006.jpg]

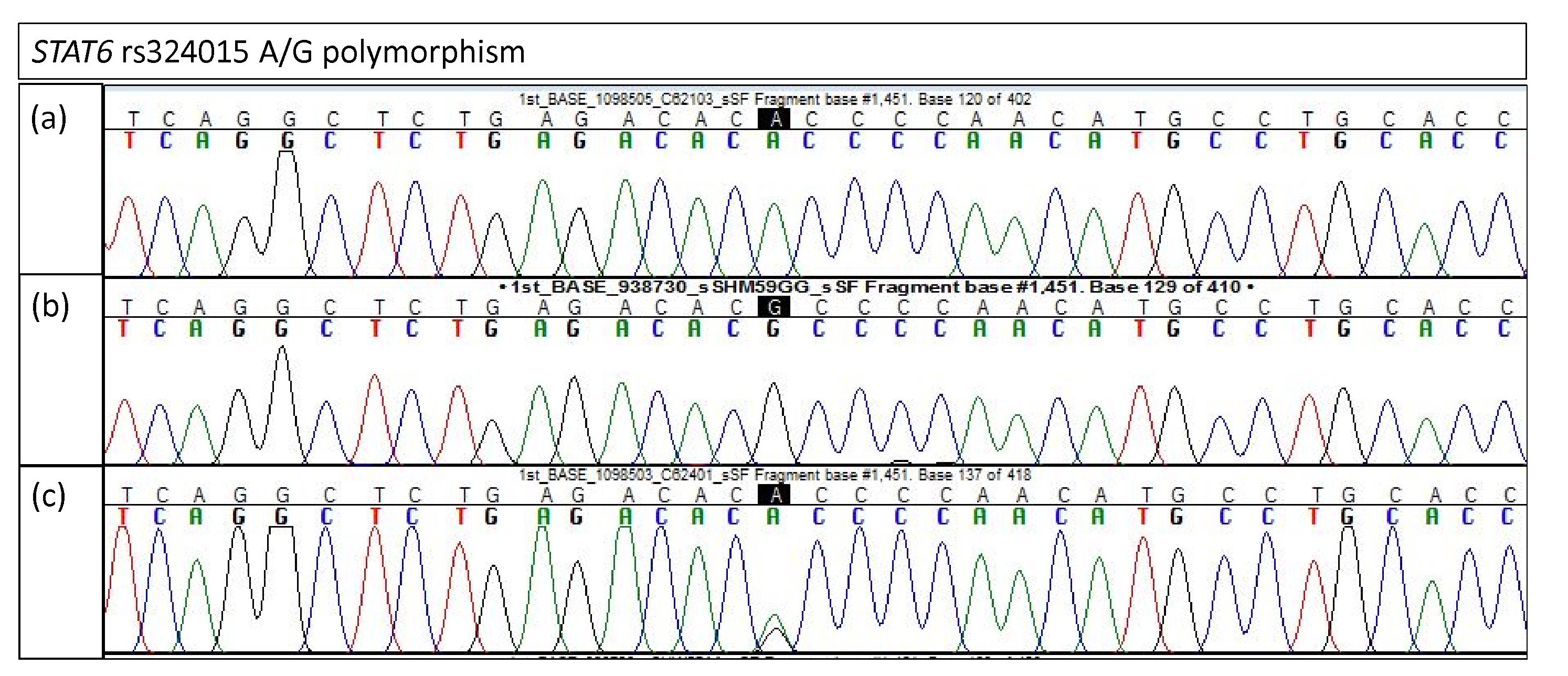

Supplement: Figure S7 — (A) Homozygous A/A (B) Homozygous G/G (C) Heterozygous A/G. [file peerj-04-1843-s007.jpg]

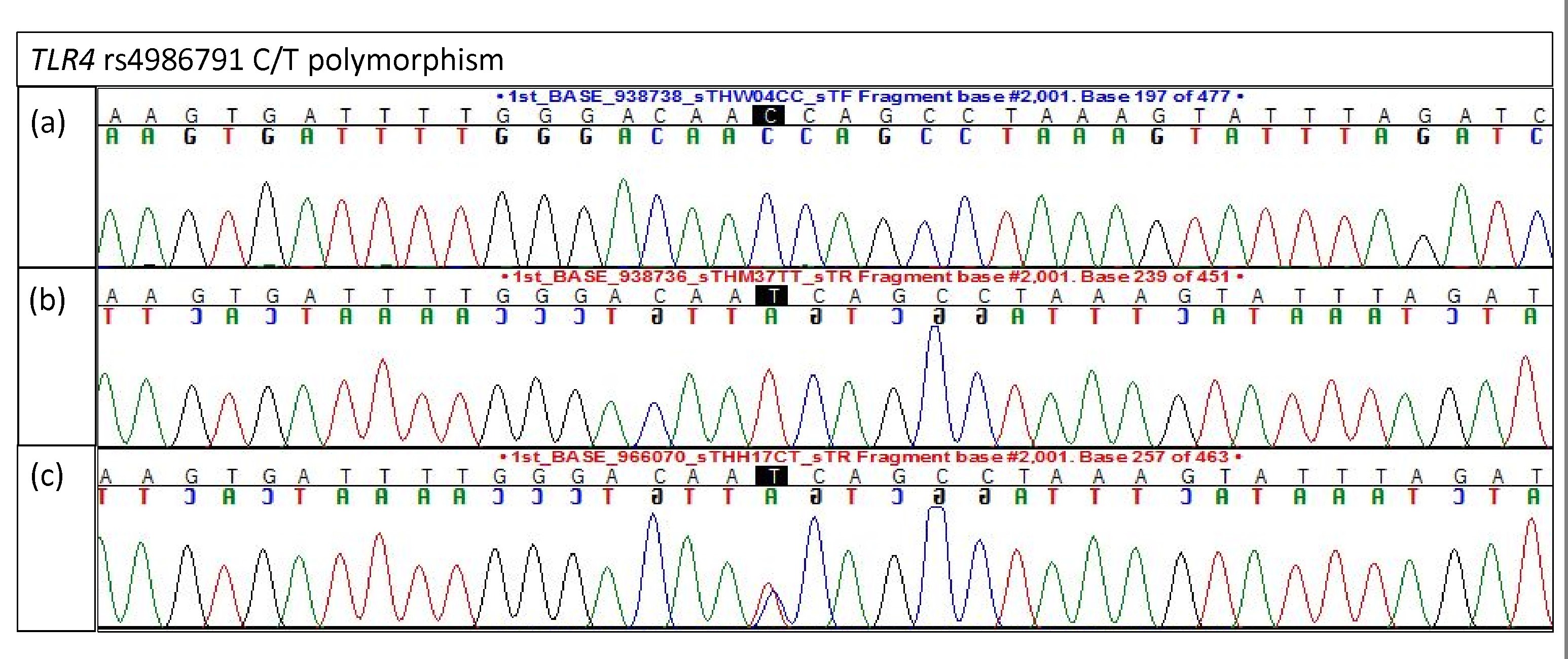

Supplement: Figure S8 — (A) Homozygous C/C (B) Homozygous T/T (C) Heterozygous C/T. [file peerj-04-1843-s008.jpg]
